# The Role of Herpes Viruses in Pulmonary Fibrosis

**DOI:** 10.3389/fmed.2021.704222

**Published:** 2021-07-22

**Authors:** Anna Duckworth, Hilary J. Longhurst, Jane K. Paxton, Chris J. Scotton

**Affiliations:** ^1^College of Medicine and Health, University of Exeter, Exeter, United Kingdom; ^2^Department of Medicine, University of Auckland, Auckland, New Zealand; ^3^Dyskeratosis Congenita (DC) Action, London, United Kingdom

**Keywords:** pulmonary fibrosis, herpes virus, CMV, EBV, telomeres, senescence, endoplasmic reticulum stress, antiviral treatment

## Abstract

Pulmonary fibrosis (PF) is a serious lung disease which can result from known genetic or environmental exposures but is more commonly idiopathic (IPF). In familial PF (FPF), the majority of identified causal genes play key roles in the maintenance of telomeres, the protective end structures of chromosomes. Recent evidence suggests that short telomeres may also be implicated causally in a significant proportion of idiopathic cases. The possible involvement of herpes viruses in PF disease incidence and progression has been examined for many years, with some studies showing strong, statistically significant associations and others reporting no involvement. Evidence is thus polarized and remains inconclusive. Here we review the reported involvement of herpes viruses in PF in both animals and humans and present a summary of the evidence to date. We also present several possible mechanisms of action of the different herpes viruses in PF pathogenesis, including potential contributions to telomere attrition and cellular senescence. Evidence for antiviral treatment in PF is very limited but suggests a potential benefit. Further work is required to definitely answer the question of whether herpes viruses impact PF disease onset and progression and to enable the possible use of targeted antiviral treatments to improve clinical outcomes.

## Introduction

A role for herpes viruses in pulmonary fibrosis remains controversial. Herpes viruses are DNA viruses and include herpes simplex (HSV) 1 & 2, Epstein Barr Virus (EBV), cytomegalovirus (CMV) and human herpes viruses (HHV6, HHV7 and HHV8 or Kaposi's Sarcoma-associated virus). Herpes viruses are ubiquitous; HSV affects up to 95% (1), EBV up to 90% (2) and CMV about 60% (3) of European populations, its prevalence rising with age. In contrast, other herpes viruses, such as HHV8 have high prevalence only in certain populations, such as Sub-Saharan Africans (4). Once herpes virus is established in a host, infection becomes latent and persists for life. Latent virus localizes within the nucleus and generally persists in episomal form, periodically reactivating as lytic virus, with different cell tropism and protein expression.

Lytic viral expression is often subclinical but may also be responsible for typical clinical manifestations such as cold sores (HSV1) or infectious mononucleosis (EBV or CMV), or in severely immunosuppressed people necrotising retinitis, encephalitis or pneumonitis (5). Periodic reactivation is universal but frequency and clinical consequences depend on multiple genetic and environmental factors, many of which are poorly understood. Herpes viruses can also integrate into chromosomal DNA and herpes viral DNA can be found in around 1% of people (6). In some cases, this integrated material can reactivate, raising the possibility of functional consequences (7).

Pulmonary fibrosis (PF) is a feature of the interstitial lung diseases, marking a progressive disorder characterized by alveolar-interstitial inflammation, accumulation of extracellular matrix, distortion of normal architecture and impairment of gas exchange. Slow deterioration in lung function is interspersed with acute exacerbations; these episodes of rapid decline in lung function, associated with high mortality, may or may not be associated with triggers such as infection. For Idiopathic PF (IPF), the most common form of PF, overall mortality is high with median survival of 3 years from diagnosis (8). Familial PF (FPF) has an established association with telomere maintenance (9) and evidence for a causal role for short telomeres in IPF is also growing (10, 11). Many groups report increased prevalence of herpes virus in IPF, FPF and animal models of PF (12–25) although this is not a universal finding (26–30).

Some herpes viruses appear able to augment other insults to the lung epithelium in causing and exacerbating fibrosis from both the lytic and latent states. In addition to the established role of these viruses in destruction of cells, there appear to be several potential herpes virus-associated mechanisms for enhancement of telomere shortening in alveolar epithelial cells (AEC). These include: induction of a pro-fibrotic cytokine environment; virally-induced endoplasmic reticulum stress which induces inflammatory signaling via NFκB and AP-1 pathways; enhanced cell proliferation regulated by viral DNA; an influence over telomeric repeat-containing RNA (TERRA) regulation on both maintenance of telomere integrity and degradation of shelterin; and a possible impact on two of the key genes implicated in FPF via the GA binding protein GABP. Given the recognized significance of telomere shortening in IPF, described in more detail below, it would be reasonable to postulate a potential role for herpes viruses in contributing to telomere attrition.

## Epidemiologic Evidence

Herpes viruses may have different roles in PF, depending on whether they are latent (DNA viral genome only) or lytic. Various studies have looked at possible roles for herpes viruses in the initiation, maintenance and acute exacerbation of pulmonary fibrosis. The results of studies in humans are summarized in Table 1.

**Table 1:** Studies of potential role of herpes viruses in pulmonary fibrosis: human.

**References**	**Virus**	**HHV type**	**Sample**	**Method**	**Patients**			**Controls**			**AGE**,	**GENDER**,				**Immuno-suppression**	
					**Positive**	**Total**	**%**	**Positive**	**Total**	**%**	**y**	**Male**	**Female**	**Smoking**	**Total**	**Yes**	**Total**
**Modified table from Sheng review (****14****)**																	
Lasithiotaki et al. (31)	HSV-1	alpha	Lung	PCR	1	11	9.1	0	4	0.0	NA	NA		NA	NA	NA	
	HHV-6	beta	BALF	PCR	5	13	38.5	8	13	61.5	64.95	18	8	14	26	NA	
	CMV/HHV7/8	beta/gamma	Lung/BALF	PCR	0	24	0.0	0	17	0.0		18	8	14	26	NA	
Lok et al. (19)	EBV	gamma	Lung	PCR and IHC	8	14	57.1	0	19	0.0	55.55	19	14	NA		NA	
Calabrese et al. (32)	EBV	gamma	Lung	PCR and IHC	13	55	23.6	0	14	0.0	52.00	47	30	53	77	NA	
	HHV-6	beta	Lung	PCR and IHC	7	55	12.7	1	14	7.1	52.00	47	30	53	77	NA	
	CMV	beta	Lung	PCR and IHC	4	55	7.3	2	14	14.3	52.00	47	30	53	77	NA	
	Parvovirus B19		Lung	PCR and IHC	0	55	0.0	1	14	7.1	52.00	47	30	53	77	NA	
Tang et al. (15)	EBV	gamma	Lung	PCR	21	33	63.6	3	25	12.0	52.41	32	18	NA		30	45
	CMV	beta	Lung	PCR	7	33	21.2	0	25	0.0	52.41	32	18	NA		30	45
	HHV-7	beta	Lung	PCR	14	33	42.4	6	25	24.0	52.41	32	18	NA		30	45
	HHV-8	gamma	Lung	PCR	15	33	45.5	2	25	8.0	52.41	32	18	NA		30	45
Folcik et al. (17)	HVS	alpha	Lung	ISH and IHC	21	21	100.0	0	21	0.0	61.30	29	13	NA		NA	
Tang et al. (33)	CMV	beta	Lung	PCR	7	23	30.4	0	19	0.0	NA	NA		NA		NA	
	EBV	gamma	Lung	PCR	15	23	65.2	2	19	10.5	NA	NA		NA		NA	
	HHV-7	beta	Lung	PCR	9	23	39.1	2	19	10.5	NA	NA		NA		NA	
	HSV-1/2	alpha	Lung	PCR	10	23	43.5	1	19	5.3	NA	NA		NA		NA	
	VZV/HHV-6	alpha/beta	Lung	PCR	0	23	0.0	0	19	0.0	NA	NA		NA		NA	
Tsukamoto et al. (16)	EBV	gamma	Lung	PCR	24	25	96.0	15	19	78.9	57.33	25	9	21	29	0	29
Stewart et al. (34)	EBV	gamma	Lung	PCR	13	27	48.1	4	28	14.3	55.47	37	18	37	55	16	27
Bando et al. (35)	Torque Teno V		Serum	PCR	55	77	71.4	138	142	97.2	65.29	147	52	153	199	0	199
Pozharskaya et al. (36)	EBV	gamma	Lung	PCR	9	13	69.2	0	4	0.0	NA	NA		NA		NA	
Wootton et al. (37)	Torque Teno V		BAL	PCR	12	83	14.5	7	29	24.1	64.06	87	25	80	112	NA	
Pulkkinen et al. (38)	EBV	gamma	Lung	PCR	11	12	91.7	0	11	0.0	58.71	18	3	11	21	2	21
	HHV-6B	beta	Lung	PCR	10	12	83.3	3	11	27.3	58.71	18	3	11	21	2	21
	HHV-7	beta	Lung	PCR	2	12	16.7	2	11	18.2	58.71	18	3	11	21	2	21
	CMV	beta	Lung	PCR	2	12	16.7	0	11	0.0	58.71	18	3	11	21	2	21
	HSV-1	alpha	Lung	PCR	1	12	8.3	0	11	0.0	58.71	18	3	11	21	2	21
	HSV-2	alpha	Lung	PCR	1	12	8.3	0	11	0.0	58.71	18	3	11	21	2	21
Lawson et al. (39)	EBV	gamma	Lung	IHC	8	23	34.8	0	10	0.0	NA	NA		NA		NA	
	CMV	beta	Lung	IHC	8	23	34.8	0	10	0.0	NA	NA		NA		NA	
	HHV8	gamma	Lung	IHC	2	23	8.7	0	10	0.0	NA	NA		NA		NA	
Kelly et al. (40)	EBV	gamma	Serum	PCR	23	27	85.2	41	50	82.0	52.25	15	11	NA		36	77
Miyake et al. (41)	Hepatitis C Virus		Medical history	NA	7	104	6.7	4	60	6.7	NA	149	15	129	164	NA	
Dworniczak et al. (27)	CMV	beta	Serum; blood leukocytes; BAL cells	PCR	12	16	75.0	11	16	68.8	38.85	21	11	14	32	0	32
Rabea et al. (42)	Hepatitis C Virus		Serum	ELISA	9	30	30.0	17	60	28.3	50.55	38	52	19	90	NA	
Santos et al. (43)	Measles Virus		Lung	IHC	2	13	15.4	3	24	12.5	61.16	18	19	NA		NA	
	CMV	beta	Lung	IHC	1	13	7.7	4	24	16.7	61.16	18	19	NA		NA	
Ushiki et al. (44)	Respiratory Syncytial Virus B		BAL	PCR	1	7	14.3	0	7	0.0	69.50	11	3	NA	NA	1	14
	CMV	beta	BAL	PCR	0	7	0.0	2	7	28.6	69.50	11	3	NA		1	14
Saraya et al. (45)	HHV-7	beta	Sputum; BALF; nasal swab	PCR	3	27	11.1	5	51	9.8	74.65	46	32	42	78	73	78
	CMV	beta	Sputum; BALF; nasal swab	PCR	2	27	7.4	2	51	3.9	74.65	46	32	42	78	73	78
	Influenza AH3		Sputum; BALF; nasal swab	PCR	0	27	0	3	51	5.8824	74.65	46	32	42	78	73	78
	Influenza H1N1		Sputum; BALF; nasal swab	PCR	0	27	0	1	51	1.9608	74.65	46	32	42	78	73	78
	Human parainfluenza virus		Sputum; BALF; nasal swab	PCR	1	27	3.7	1	51	2.0	74.65	46	32	42	78	73	78
	Human metapneumovirus		Sputum; BALF; nasal swab	PCR	0	27	0.0	1	51	2.0	74.65	46	32	42	78	73	78
**Further papers mentioned in our review**																	
Egan et al. (46)	EBV	gamma	Lung	IHC	14	20	70.0	2	21	9.5	59.50	31	10	31	41	14	41
Manika et al. (27)	EBV	gamma	BAL, serum	IHC, PCR	10	17	58.8	11	50	22.0							
Magro et al. (24)	CMV	beta	Serum		9	18	50.0										
Vergnon et al. (23)	EBV	gamma	Serum		10	13	76.9	0	12	0.0							
Yin et al. (29)	EBV	gamma	Lung	RNA-seq, PCR	0	28	0.0	0	20	0.0							
	Hepatitis C Virus		Lung	RNA-seq, PCR	0	28	0.0	0	20	0.0							
	HVS	alpha	Lung	RNA-seq, PCR	0	28	0.0	0	20	0.0							
Le Hingrat et al. (30)	HSV-1/HSV-2/VZV/HHV-8	alpha/gamma	Lung	PCR	0	19	0.0	0	9	0.0	51–62	23	6		29	2	28
	CMV	beta	Lung	PCR	2	19	10.5	1	9	11.1	51–63	23	6		29	2	28
	HHV-6	beta	Lung	PCR	12	19	63.2	4	9	44.4	51–64	23	6		29	2	28
	EBV	gamma	Lung	PCR	12	19	63.2	4	9	44.4	51–65	23	6		29	2	28
	HVS	alpha	Lung	PCR	0	19	0.0	0	9	0.0	51–66	23	6		29	2	28
	Torque Teno V		Lung	PCR	12	18	66.7	9	9	100.0	51–67	23	6		29	2	28
Wangoo et al. (26)	EBV- associated RNA (EBER)	gamma	Lung	IHC, PCR	0	12	0.0	0	15	0.0							
Weng et al. (47)	HHV		NP swab AE-IPF vs stable IPF controls	RNA-seq	15	30	50.0	4	30	13.3	59–65	48	0	34	48	48	48
Zamo et al. (28)	EBV	gamma		IHC, PCR													
	HHV-8	gamma															

*Grey shading highlights those studies related to herpesviruses*.

A large meta-analysis of case-control studies showed significant association between herpes virus infection and IPF (14). Presence of herpes viruses CMV, EBV, HHV7, and HHV8 all correlated with the risk of IPF (*p* = 0.001), although interestingly, not with acute exacerbation (*p* = 0.988) where there are less data. Not all studies have shown associations between herpes viruses and PF. In particular, the evidence linking acute exacerbations with herpes viruses is limited.

Vergnon et al. were first to suggest a link, showing higher anti-EBV antibody titres and higher prevalence of EBV VCA (viral capsid antigen) IgA in serum of IPF patients compared with controls with PF of known cause (23). Egan and colleagues found EBV lytic phase protein expression (gp340/220 and viral capsid antigen, VCA) on immunohistochemistry of lung biopsies from 14 of 20 patients with IPF, including 9 of 14 who were not on immunosuppressive drugs compared with 2 of 21 control lung biopsies; *p* = 0.0001 (46). In agreement with earlier studies, Tsukamoto et al. showed EBV latent membrane protein (LMP) by PCR in AEC in 24 of 25 patients with idiopathic and 5 of 5 with systemic sclerosis-associated PF (SSc), compared with 10 of 14 controls. The detection ratio was higher in IPF patients than controls (*p* = 0.047). Immunohistochemistry showed LMP protein in 9 of 29 patients with IPF, but no immunoreactivity in SSc (with PF) or healthy control biopsies. LMP detection was significantly associated with PF itself and with disease progression (*p* = 0.022) (16).

A study in 2003 found that lung tissue from 97% of PF patients (8 FPF and 25 IPF) contained PCR-amplifiable DNA from at least one Herpes virus, compared with 36% of controls (*p* < 0.0001) (15). The dominant virus differed between IPF and FPF (EBV and CMV, respectively). Subsequent work showed CMV seropositivity with patchy CMV RNA detectable by *in situ* PCR of lung biopsies from 9 of 19 PF patients. CMV RNA detection was focal, affecting endothelial cells and alveolar macrophages (24).

Other studies again showed increased prevalence of serum EBV IgA, possibly implying a mucosal process, and EBV DNA in bronchoalveolar lavage fluid from IPF patients. Sixty percent of 17 PF patients compared with 22% of 50 healthy controls and 24% of 46 controls with other interstitial lung diseases had detectable serum EBV IgA to early, nuclear or capsid antigens (*p* = 0.01). Three antibody positive PF patients but none of the controls had detectable EBV DNA, confirmed by sequencing (*p* = 0.02) (25). The following year, Lawson et al. found EBV (LMP1), CMV (immediate, early and late) or HHV8 (K-cyclin) antigens, by immunohistochemistry (39). Herpes virus antigens were co-localized with ER stress markers, in type II alveolar epithelial cells (AEC2) in 15 of 23 PF lungs but not in 10 control lungs (*p* = 0.0005), implying a functional consequence of infection. Calabrese et al. found CMV, EBV, HHV6, 7 or 8, but not other viruses by PCR in bronchoalveolar lavage in 40% of PF vs. 7.3% of disease control, non-IPF diffuse parenchymal, lung transplant explants (*p* = 0.0003), and 0% of healthy lungs (32). EBV was the most common, accounting for 60% of IPF-related infections, but was not detected in controls. RNA *in situ* hybridization detected EBV EBER (Epstein–Barr virus-encoded small RNAs) in AEC in 40% of positive PF cases.

A 2014 investigation found DNA of *Herpesvirus saimiri*, a virus closely related to HHV8, in the regenerating epithelial cells of 21/21 IPF cases and 0/21 controls (17). The authors detected four proteins known to be encoded in the genome of several γ-herpes viruses (cyclin D, thymidylate synthase, dihydrofolate reductase, and interleukin-17) that were strongly co-expressed in the regenerating epithelial cells of each of the 21 idiopathic pulmonary fibrosis cases and not in controls. Furthermore, they showed that the source of the cyclin D RNA, a key regulator of cell proliferation in active IPF was *Herpesvirus saimiri* and not human, implying that cell cycle regulation was virally-controlled. Such virally-controlled differences in cell turnover could contribute to fibrosis, perhaps via telomere shortening and premature replicative senescence. However, in the absence of further data, this remains speculative. Although its primary host is the squirrel monkey, up to 7% of humans may be infected with *H. saimiri*, likely depending on exposure (12). Association of IPF with *H. saimiri* has not been replicated elsewhere, although, to our knowledge *H. saimiri* has not been specifically sought, except in one other study which found no herpes virus genetic material in PF patients or controls (20). Of note, squirrel monkeys are permitted as pets in some US states, and it is conceivable that this data represented a locally increased prevalence of *H. saimiri* infection, with pathological consequences for some of those infected.

Among the negative studies, that of Yin et al. is most compelling: they looked specifically, by RNA next generation sequencing, at viral RNA and found no increase of herpes virus gene transcripts in 28 IPF lung transplant explants from different sources, compared with 20 healthy lungs. However, they did find some evidence of human endogenous retrovirus (HERV)-K, the expression of which is potentiated by EBV. Since only 1 EBV-associated RNA (EBER) was sought it remains possible that this latency-associated gene is only expressed at levels below the limit of detection in infected lung cells (29). A very recent study from Le Hingrat et al. found a similarly high prevalence of herpes viruses in both groups of 19 IPF and 9 control lung samples and no evidence of *H. saimiri* in either group (30). Likewise earlier studies have failed to show any evidence of EBER RNA by *in situ* hybridization in PF lungs (26). A study of CMV failed to show difference in seroprevalence or bronchoalveolar lavage copy number between 16 untreated PF patients and 16 healthy volunteers, although leukocyte CMV copy number was higher in PF patients (27). HHV8 and EBV proteins NCL-HHV8-LNA, LMP1, EBV RNA (EBER) by immunohistochemistry and EBV DNA by PCR were not found in IPF lung biopsies in another study (28), and HSV1 and EBV were not detectable by PCR of pan-microarray, generated from bronchoalveolar lavage samples from 40 Japanese or Korean patients with stable IPF (37).

## Acute Exacerbations In Pulmonary Fibrosis

Since patients in the studies discussed above were stable at the time the lung material was obtained, the results cannot address any role for herpes viruses in acute exacerbation, including subclinical exacerbation, which may nevertheless contribute to functional decline. Investigation of acute exacerbation has necessarily been limited by barriers in obtaining suitable material in very sick patients. Acute exacerbations were previously defined as deterioration in the absence of infection or other known factor. The role of infection, often cryptic, in triggering acute decline is now well-accepted but the definition has made it difficult to assess the role of virus in such decline.

In 2010, Huie et al. noted the presence of CMV or HSV by PCR or culture in bronchoalveolar lavage of 4 of 27 patients with IPF and other fibrotic diseases. In the patients who had biopsy material available, no CMV or other viral inclusions were seen, nor was there detectable viraemia, suggesting that the viruses were either commensals, or at least were not exerting any direct cytopathic effect (48).

Wootton et al. investigated bronchoalveolar lavage samples from PF patients undergoing acute exacerbation and found PCR, array or deep sequencing evidence of virus presence in only 19 of 43 cases, of which 3 were herpes viruses (EBV in 2 cases and HSV in 1) (37). Ushiki et al. examined bronchoalveolar lavage fluid from 14 patients with acute exacerbations of interstitial lung disease for CMV by PCR. Seven patients fulfilled the criteria for IPF and the others were classified as idiopathic interstitial pneumonia, but did not fulfill CT criteria for IPF; one of the latter was taking immunosuppressive drugs. Two patients, neither of whom had definite IPF, had detectable CMV (44). Given that the control group here may have had earlier stages of IPF, it is difficult to draw firm conclusions. Weng et al. detected HHV in 15 of 30 nasopharyngeal swabs from subjects experiencing an acute exacerbation of IPF (AE-IPF) vs. four of 30 individuals with stable IPF (47), while Santos et al. detected CMV infection in over a third of histological sections from 37 AE-IPF subjects (43). In a recent review discussing the contribution of infection and the microbiome in acute exacerbations of IPF, Invernizzi and Molyneaux provide a further summary of the studies cited here, linking HHV infection to AE-IPF (49).

Overall epidemiological evidence appears to be in favor of a role for herpes viruses, at least in stable disease (14), but a lack of data, poorly defined control groups and the exclusion of those with overt infection makes it difficult to draw any conclusion on the role of herpes viruses in acute exacerbations (14). Apparent inconsistencies between studies, particularly in the prevalence and types of herpes virus are troubling. These differences may have arisen because of the patchy nature of herpes virus infection, as a result of different handling of the technical approaches (50), differences in and limited range of the herpes virus DNA, RNA, proteins, or serology assessed and/or major differences in patient/control location, clinical status and material. Ethnicity may also influence the prevalence of exacerbations (51) and since host genetics influences the immune response to herpesviruses [e.g., (52)] and most of the genetic variation is ethnic and population specific, these influences also need to be controlled. Newer next generation techniques, such as used by Yin et al., are likely to be more sensitive than older methods. Development of less invasive techniques with methods that sample a wider lung region and larger patient numbers, and conversely analytical techniques that provide cell-level data, but require invasive sampling, will be important in resolving these conflicting data and answering the question as to whether herpes viruses are important in IPF. Collaborative initiatives such as the IPF cell atlas maximize the benefit of these difficult-to-obtain data (53).

## Asymptomatic Relatives At Risk Of FPF

An extensive study of asymptomatic relatives of patients with FPF showed that patients with PF had increased prevalence of herpes virus DNA in cell-free bronchoalveolar lavage fluid and evidence of herpes virus antigen expression in AEC (18); asymptomatic relatives also had elevated herpes virus DNA levels, overlapping with control and PF groups. These data suggest that herpes virus infection may precede onset of symptomatic pulmonary fibrosis. Alternatively, if PF patients were more permissive to herpes virus infection they might be more likely to infect relatives. Longitudinal data are required to address this question.

## Herpes Virus In Animals

In contrast, observations in other animals provide more compelling evidence of a role for herpes virus in PF (19, 21, 54). The results of studies in animals are summarized in Table 2. Spontaneously acquired herpes virus is associated with PF in a variety of domestic mammals (20). Experimental infection of free living, outbred horses with a native horse herpes virus induced pulmonary fibrosis in 3 of 5 horses infected, compared with neither of 2 sham infection control horses (21). Other studies found that gammaHV-68 induced fibrosis in aged mice (55, 56), in STING gain-of-function mice (61) and in mice subject to sub-fibrotic bleomycin challenge (19). Mice with established PF experienced histological and clinical exacerbation when challenged with herpes virus (57, 58, 62). All of these studies establish mechanistic links between herpes virus infection of the lung and the subsequent development of PF.

**Table 2:** Studies of potential role of herpes viruses in pulmonary fibrosis: animal models.

**References**	**Virus**	**Model**	**Method**	**Measure**		**Cases**			**Controls**					
					**Treated**	**Cases**	**Total**	**%**	**Controls**	**Total**	**%**	**Age, y**	***p*-value**	**Comments**
**Animal studies mentioned in our review**														
Lok et al. (19)	Murine γHV-68	Bleomycin resistant mouse	Treatment with virus followed by bleomycin	Fibrosis and inflammation in lung via IHC/Collagen	14				16			5–6 wks	<0.001	
Williams et al. (20)	Equine γHV EHV-5	Detection of virus in horses with equine multinodular pulmonary fibrosis	Nested PCR	EHV-5 DNA in lung		24	24	100.0	0	23	0.0	4–28 y		
	Equine γHV EHV-2	Detection of virus in horses with equine multinodular pulmonary fibrosis	Nested PCR	EHV-2 DNA in lung		8	24	33.3	1	9	11.1			
Willliams et al. (21)	Equine γHV EHV5	Clinically normal horse	Inoculation with virus into lung	Increase in collagen content in alveolar region	6	5	6	83.3	0	2	0.0	10–26 y	*p <* 0.001	Horses were selected from 22 horses for the study based on having the lowest titers of antigen-binding antibody.
Naik et al. (55)	Murine γHV-68	Young healthy mice	Intranasal infection	Increase in collagen content and fibrosis	5				3			4 months	not sig	
	Murine γHV-68	Aged healthy mice	Intranasal infection	Increase in collagen content and fibrosis	5				3			15–18 months	0.0003	
Torres-González, et al. (56)	Murine γHV-68	Young healthy mice	Intranasal infection	Increase in collagen content and fibrosis	8							2–3 months	*p <* 0.05, *n* = 3	
	Murine γHV-68	Aged healthy mice	Intranasal infection	Increase in collagen content and fibrosis	5							≥18 months		
McMillan et al. (57)	Murine γHV-68	Flourescin isothiocyanate-induced lung fibrosis mice	Intranasal infection with virus or saline	Increase in collagen	10–12				10–12			6–8 wks	*p <* 0.001	
Ashley et al. (58)	Murine γHV-68	Bleomycin-induced lung fibrosis mice	Intranasal infection with virus or H1N1 influenza	Increase in collagen	3–6				3–6				*p <* 0.05	Evidence suggested that lytic viral reactivation was required and infection with CMV (a β herpesvirus) did not exacerbate bleomycin induced fibrosis
Vannella et al. (59)	Murine γHV-68	Mice with established latent herpesvirus infection vs. controls with mock infection	Fibrotic challenge with fluorescin isocyanate	Increase in collagen	12				14			2–5 months	*p <* 0.001	Evidence showed that latent infection could induce infection even when the subsequent insult is insufficient on its own and that lytic reactivation following the insult is not required.
Stoolman et al. (60)	Murine γHV-68	Young healthy mice	Intranasal infection with virus or mock and 28 d delay for virus to become latent	ELISA for cytokines and chemokines from AM cells extracted by BAL	6				6			2–4 months	*p <* 0.001	AMs are infected and express more transforming growth factor TGF-β1 (*P* < 0.0001), TNF-α (*P* < 0.001), CCL2 (*P* < 0.0001), CCL12, and IFN-γ (*P* < 0.0001) compared with AMs from mock-infected mice.
	Murine γHV-69	Young healthy mice	Intranasal infection with virus or mock and 28 d delay for virus to become latent	Lung tissue supplemented with cidofovir to prevent viral reactivatin and cell lysis	8				8			2–4 months	*p <* 0.05	TGF-β1 (*P* < 0.0001), CCL12 (*P* < 0.0001), and TNF-α (*P* < 0.05) were expressed at significantly higher levels by mesenchymal cells harvested from γHV-68-infected mice compared with mesenchymal cells from mock-infected mice. Results showed that mesenchymal cells, and CD19-enriched cell populations from the lung and spleen express M3 and/or glycoprotein B (gB) viral mRNA and harbor viral genome.

## Potential Mechanisms Of Herpes Virus-Induced Damage

### Direct Damage

Respiratory viruses can cause damage via direct cytopathic effects, by potentiation of secondary bacterial infections or via associated inflammation (12, 63). Presence of detectable infection is associated with higher short-term mortality in patients with critical illness (64–66). Lytic herpes viruses are thought to cause pulmonary fibrosis in baboons and cats (54, 67). Mouse models have shown alveolar damage and PF exacerbation in the presence of lytic γHV (19, 57, 58, 61). Herpes viruses have cytopathic effects when in the lytic/reactivation phase (68–70) and EBV is known to replicate in type 2 alveolar cells of PF patients (46). While single, mild or infrequent pulmonary infection typically heals without scarring, severe or persistent infection is often associated with aberrant healing and structural damage, including fibrosis. EBV virus can produce Zta protein in the lung, which enhances production of MMP9, damaging alveoli and allowing increased penetration of inflammatory cells (71). Increased prevalence of persistent viral infection and age-related waning of immunity might therefore predispose to more fibrogenic outcomes after viral infection, consistent with the observed increasing incidence of PF in older people. However, not all viruses are fibrogenic and some may even be protective: respiratory syncytial virus (RSV) can protect mice from vanadium pentoxide-induced fibrosis (72).

The microbiome is increasingly recognized as an important modulator of disease, including in pulmonary fibrosis (12, 73, 74) - although data concerning the pulmonary virome is lacking, as is understanding of the mechanisms whereby the microbiome exerts its influence. However, the recognition that the lung is not a sterile environment and that extensive cross-talk exists between lung, gut and other organs opens up a whole area of fruitful enquiry [e.g., (75)].

### Direct Damage Alone Is Insufficient to Account for Herpes Virus-Associated Pulmonary Fibrosis

Direct herpes virus infection cannot be the only factor in induction of PF. Herpes viruses are common, whereas pulmonary fibrosis is not, implying an essential role for other genetic or environmental factors in its aetiology. Moreover, animal models suggest that latent herpes virus can induce PF with appropriate genetic or environmental background. Murine γHV-68, a γ-herpes virus analogous to EBV, *H. saimiri* or HHV8 (76), given to mice between 14 and 70 days prior to an otherwise subclinical fibrotic insult, causes fibrosis and increased collagen deposition (59). This work expanded on the earlier studies by Lok et al. who noted exacerbation of fibrosis in bleomycin-resistant mice infected with γHV68 7 days prior to bleomycin challenge. Whereas, Lok hypothesized that lytic herpes-virus infection was likely to be responsible, other work demonstrated induction of PF even after resolution of lytic infection, or when a modified γHV-68 incapable of lytic infection was used (19, 60). Subsequent studies showed that mice experienced increased fibrotic response even if the herpes challenge was administered after the fibrotic insult, a finding of potential importance to the human situation, where fibrotic insults, such as smoking or environmental pollution may well-precede the herpes virus infection (60).

### Modulation of Cytokine Release and the Pro-fibrotic Environment

AEC2, alveolar macrophages, neutrophils, fibroblasts and T cells all contribute to the fibrogenic environment in PF. Repeated environmental alveolar micro-injury, in the context of a Th2 (IL4, IL13) or Th17 (IL6, TGFβ)-polarized lymphocyte response can induce epithelial-mesenchymal transition of AEC2, their differentiation to myofibroblasts, activation of a type 2 cytokine-mediated immune response, profibrotic macrophages, fibroblast-myofibroblast transformation and neutrophil influx, in a complex manner which incorporates several paracrine amplification loops. Extracellular matrix, such as collagen, hyaluran or fibronectin, laid down by myofibroblasts causes architectural distortion and impaired gas transfer of clinical pulmonary fibrosis, and will itself contribute to fibrotic progression (77, 78). TGFβ is a key cytokine in induction of fibrosis and is itself found at higher levels in the presence of herpes virus. Increased TGFβ expression has been detected in alveolar macrophages and metaplastic AEC of human PF lung tissue, including in association with herpes viruses (32). Vascular endothelial growth factor (VEGF) may also play a role. VEGF is expressed by hypoxic cells, including alveolar epithelia, macrophages, lymphocytes and fibroblasts. VEGF is overexpressed in PF, with reports suggesting both damaging and protective/compensatory roles for differentially spliced isoforms (79).

Chemokines, particularly CCL2 (MCP1) mRNA and protein, as well as platelet-derived growth factor (PDGF), are expressed by alveolar epithelium in IPF but not normal human lung biopsies (80). CCL2 attracts fibroblasts and “alternatively-activated” M2 macrophages, which promote fibrosis via secretion of cytokines, particularly TGFβ (77, 81, 82). Only one human study has sought to link herpes viruses to potentially profibrotic cytokines. *H. saimiri* is known to promote expression of human IL-17. Folcik et al. demonstrated co-expression of *H. Saimiri* and IL-17 in regenerating AEC2 from human PF lung biopsies (17).

Animal studies demonstrate association of herpes viruses with a pro-fibrotic immunological environment. γHV-68 infection of Th2-biased mice increases secretion of TGFβ and cysteinyl leukotriene synthesis in AEC2 (59), hyperplasia of AEC2, increased myofibroblast transformation, higher levels of MMP7 and increased collagen deposition, with decline in forced vital capacity and airway remodeling (83), all features typical of PF. As well as its direct role in pulmonary fibrosis, TGFβ has multiple other effects, including an immunosuppressive, tolerogenic role, and is associated with increased susceptibility to γHV in mouse models (84), thus creating a potentially deleterious feedback loop between local herpes virus infection and TGFβ production.

Another study showed that aged, but not younger mice developed γHV-associated pulmonary fibrosis, with TGFβ production by alveolar macrophages and AEC2 and increased sensitivity of fibrocytes to TGFβ, despite similar levels of viral DNA and Th1/Th2 cytokine ratio (55). Use of aged mice may be particularly relevant to the observed human situation, where herpes virus infections are contracted throughout life, but pulmonary fibrosis is largely a disease of the elderly.

In a Th2-biased (IFNγ knockout) mouse model, PF was initiated by γ-herpes virus infection and was associated with high levels of lung VEGF, which were reduced when lytic infection was controlled or prevented by infection with lytic-phase-incompetent virus (81).

Alveolar macrophages and other immune cells in γHV-68-infected mice also produce chemokines such as CCL3, 4 & 5 (85), and more importantly CCL12, even after resolution of lytic phase. CCL12 has high homology with human MCP1/CCL2, known to be profibrotic (59). Thus, the available evidence implies that lytic herpes virus infection is not always required for initiation of PF. Latent infection is also associated with AEC2 production of cysteinyl leukotrienes, TNF and TGFβ. Leukotriene D4 increases TGFβ production in human lung epithelial cells, attracts profibrotic monocytes and neutrophils, and can cause fibrocyte/blast proliferation, with increased collagen production (86).

Two mouse studies demonstrate relevance of γHV as just one of multiple cofactors in the development of PF. Latent γHV infection post allogeneic transplant in a Th1-biased mouse model was associated with pneumonitis and pulmonary fibrosis, despite similar γHV levels in transplanted and untransplanted control mice, who did not develop PF. The PF was associated with increased TGFβ of non-lymphocyte, presumed lung, origin. Alternatively, activated macrophages, with a profibrotic, TGFβ-secreting phenotype were prominent (84). Severity of PF correlated with previous levels of lytic virus, implying that prior structural damage caused during the lytic phase might be important (84). Cook et al. *s*howed that pulmonary CMV reactivation was universal when Th2-biased mice were subject to sepsis induced by caecal puncture. CMV reactivation under these circumstances was associated with increased and prolonged secretion of TNFα, IL1β, neutrophil chemokine KC and MIP2/CCL4 with collagen deposition and pulmonary fibrosis, which did not occur in uninfected mice (87). A requirement for coexistence of multiple profibrogenic factors would be consistent with the development of pulmonary fibrosis in only a fraction of those infected with herpes viruses.

Acute PF exacerbations may require different cytokine stimuli: a mouse model of γHV PF (57) demonstrated that acute lytic γHV induced pulmonary fibrosis. PF is usually associated with a Th2 environment, but this model demonstrated that herpes virus infections could produce fibrosis and acute clinical decline, reminiscent of acute PF exacerbation, even in Th1-biased, and Th2-knockout (IL4/IL13^−/−^) mice. As well as increased AEC and macrophage CCL2/12 production, there were increased levels of the type I cytokines, IFNγ and TNFα. Type 1 cytokines are usually considered protective against PF, but, as illustrated by this model, may be associated with acute alveolar damage and be deleterious early in the course of an exacerbation, particularly since TNFα can induce production of profibrotic cytokines such as TGFβ and PDGF (88). Of note, a double-blind randomized clinical trial of etanercept, an anti-TNF agent, did not show significant differences in progression in patients with PF after 48 weeks (89).

Lytic, but not latent γHV infection also resulted in pulmonary fibrosis in Th1-biased, STING gain of function mice. STING mutations are associated with increased production of type 1 antiviral cytokines, IFNγ and IFNβ, and in humans are associated with SAVI, an autoinflammatory disorder, known to be triggered by viral infection. This work further hints at a possible role of Th1 cytokines as mediators in initiation of acute virally-induced PF exacerbations (61). Th17 pathways were implicated in another mouse bleomycin model of acute exacerbation, where intra-tracheal HSV1 was found to induce ER stress and acute lung injury via an IL17-dependent pathway. HSV1-exposed mice showed high mortality compared with saline-challenged mice (62).

A role in exacerbations may be γHV-specific, since CMV, a betaherpes virus, and non-herpes viruses influenza, MAV-1 or the bacterium *Pseudomonas* were not associated with exacerbation in another mouse model (58).

Thus, with respect to modulation of cytokines, there exists a body of evidence suggesting that the presence of lytic or latent herpes viruses can play a range of key roles in creating a cell signaling environment conducive to the development of fibrosis from prior or subsequent lung injury.

### Pulmonary Hypertension and Remodeling

Herpes virus infection may exacerbate other PF-associated pathologies. Pulmonary hypertension is common in PF, affecting up to 40% of patients (90) and associated with worse outcomes (91). Gamma herpes viruses are vasculotropic and there is some evidence of their direct involvement in pulmonary hypertension (32). Herpes virus, particularly EBV, in explant lungs of patients with PF undergoing transplantation was associated with vascular remodeling. There was increased TGFβ expression and arterial intimal thickening, compared with disease and healthy control lungs, along with higher mean pulmonary arterial pressure and worse clinical outcomes (32).

### Ageing, Telomere Attrition, and Cellular Senescence

Alveolar injury results in AEC2 proliferation and increased expression of telomerase activity in healthy lung (92). Telomeres are non-protein-coding nucleotide sequences at the end of each chromosome, stabilized by their looped tertiary structure and telomere-related protein complexes (“shelterins”). With each cell division, telomere length becomes progressively shorter, until a critical length is reached whereby the host cell becomes apoptotic, senescent or potentially undergoes malignant transformation. Telomerase lengthens the telomeres and can mitigate, but not prevent, proliferation-associated telomere shortening. Thus, aged mammals tend to have shorter telomeres than young mammals, particularly in proliferating cells (93). Senescence results in dysfunctional but apoptosis-resistant cells, with a TGFβ-dominated, profibrotic cytokine profile (94). Defects of telomere repair are associated with cellular senescence and pulmonary fibrosis in humans (93, 95) and pulmonary fibrosis is predominantly a disorder of older people (96).

In families with multiple cases of PF the majority of causal variants found to date, occur in genes involved in telomere maintenance, e.g., (97). Recent evidence also supports a causal role for prematurely short telomeres in idiopathic PF. One study showed very short telomere lengths, similar in length to those found in patients with familial PF, in biopsies from the lungs of 15 of 28 IPF patients (10) and our team reported results inferring a causal effect of short telomeres in IPF using the statistical method of Mendelian randomization in large cohorts with genetic data (11). Studies in mouse models have shown that it is the AEC2 that are key to the short telomeric cause of lung remodeling and fibrosis (98) with dysfunctional chromosomal replication leading to cellular senescence and triggering the pro-fibrotic senescence-associated secretory phenotype (SASP) (99).

Herpes virus infection alone can induce pulmonary fibrosis in aged but not in young mice (which have very long telomeres), associated with increased CXCL12, a fibrocyte-attracting chemokine, and TGFβ expression (55, 56) suggesting the possibility of telomere-related senescence.

A study of 400 healthy individuals over 3 years reported a correlation between seropositivity for herpes virus infections and significant telomere attrition (with seropositivity to more herpes viruses being additively associated with greater leukocyte telomere length attrition), suggesting exposure to such infectious agents should be an important consideration in studies of telomere dynamics (100). A very recent study of 1,708 individuals aged 20–49 showed shorter telomeres in a small subgroup (3%) with latent pathogen burden characterized by high probabilities of infection with HSV1, CMV and *H. pylori* (101).

Latent herpes virus infection may impact telomere length by various mechanisms. In human diploid fibroblasts, HSV1 rearranges telomeres, is able to induce transcription of TERRA, a non-coding RNA essential for telomere homeostasis and degrades TPP1, a component of shelterin, a protein complex critical for stabilizing telomeres. Increased TERRA dampens the DNA damage response, and together with the enhanced telomere loss associated with TPP1 reduction, promotes cellular senescence, rather than apoptosis of aged cells, facilitating further HSV1 replication (102).

Human herpes virus 6A and 6B, and possibly human herpes virus 7 can integrate into telomeres, enabling somatic and sometimes vertical transmission (50). Indeed, evidence suggests that components of the shelterin complex such as TRF1 and TRF2 are recruited by the viral genome to aid chromosomal integration (103) and optical mapping highlights the way in which the viral genome hijacks and mimics the cellular telomere maintenance mechanisms (104). The presence of a large foreign genome may well interfere with telomere function, risking profibrotic cellular consequences such as senescence. Reactivation of latent telomere-integrated HHV6, is postulated to occur by one of several excision mechanisms, each of which result in loss of telomere sequence, and can therefore in some cases result in cellular telomere failure (105). In the event of a systemic trigger for HHV-6 reactivation, such as intercurrent infection, reactivation from multiple cells could trigger a clinically relevant telomere failure, perhaps resulting in acute exacerbation of pulmonary fibrosis. HHV6 reactivation itself could provide or exacerbate such a stimulus. Presence of genetic variants in helicases or shelterin components could amplify the deleterious effect of HHV-6 excision (105) and contribute to the molecular and clinical phenotypes observed in some telomere biology disorders (95).

Stem cell transplant is known to be associated with reactivation of chromosomally-integrated HHV6, e.g., (106). People with telomere biology disorders have a high prevalence of pulmonary fibrosis and other fibrotic disease after stem cell transplantation. While there are several possible mechanisms for this observation, it is feasible that reactivation of chromosomally-integrated viruses might be one factor in exacerbating the fibrotic process. Wood and Royle highlight the importance of longitudinal studies of HHV6 in those undergoing stem cell transplantation in order to address this question (105).

Cellular competition for transcription factor binding can be impacted by herpes viruses, which could decrease telomere length by “micro-competition.” After establishing a latent infection, the viral N-box (a core binding sequence contained by many viruses in their promoter /enhancer regions), binds the cellular transcription factor GA binding protein (GABP) p300 transcription complex, causing production of abnormal levels of cellular proteins. Viruses with strong N-boxes in their promoters/enhancers include herpes viruses EBV, CMV, HSV, VZV, Hepatitis B & C (HBV, HCV) and Human Papillomavirus (HPV). CMV has the strongest promoter/enhancer of these. The micro-competition theory suggests that a latent CMV binds the host GABP-binding N box, decreasing the availability of GABP to the promoter of TERF2, which encodes a shelterin protein, essential for telomere maintenance (107). Herpes virus infection-associated proliferation will further accelerate telomere loss leading to death or lack of function in AEC2 with consequent failure to replace the lost AEC1 responsible for gas exchange (56, 83). Gene set enrichment analysis of fibroblasts from rapidly progressing IPF lung compared with controls also showed that the most significantly down-regulated pathways included checkpoint signaling, DNA replication and telomere maintenance (108).

To summarize for ageing, telomere attrition and cellular senescence, there is growing evidence for the causal role of compromised telomere maintenance or short telomeres in PF pathogenesis, certainly in a substantial subset of patients. Here we present several reports of herpes virus interactions with cellular DNA that could also affect telomere maintenance.

### Endoplasmic Reticulum Stress

Ageing individuals have higher levels of endoplasmic reticular (ER) stress. Increased ER stress and apoptotic markers are evident in explants of idiopathic PF patients, perhaps related to generically shorter telomeres, higher cellular senescence and higher TGFβ levels (109, 110). CMV can activate the unfolded protein response in cultured human fibroblasts, modifying ER stress proteins to favor viral persistence (111). ER stress alone is associated with reactivation of herpes virus, raising the possibility of a deleterious feedback loop (112).

Herpes virus antigens co-localize with ER stress markers in AEC2 in IPF lung biopsies as evidenced by markers of the unfolded protein response in the AEC (39) and correspond with presence of EBV or CMV DNA in samples or AEC2 from at-risk first degree relatives of PF patients, suggesting that herpes virus infection and ER stress predate overt lung fibrosis in many cases (18).

Aged, but not young, Th1-biased mice infected with γHV developed prolonged pulmonary inflammation and PF, compared with young mice who showed resolution without long-term fibrosis. Aged mice showed marked increase of ER stress markers and apoptosis of AEC2, prior to onset of fibrosis, despite controlling herpes virus to the same extent as young mice (56). Thus, again, herpes viruses can impact a characteristic that is associated with the development and progression of fibrosis, including PF (113).

In summary, there appear to be several plausible mechanisms by which herpes viruses could also enable or promote lung fibrosis via ER stress.

## Implications For Treatment

### Available Drugs and Their Impact on Animals and Humans

Several antiviral drugs with activity against lytic herpes viruses are currently available (Val)acyclovir and (val)ganciclovir are widely available, and well-tolerated even in long-term use for treatment and prophylaxis of herpes simplex, zoster, and cytomegalovirus disease, respectively (114, 115). Foscarnet and Cidofovir are alternatives for resistant viruses, limited by toxicity and the need for parenteral administration (116, 117). After preferential conversion by viral thymidine kinases, these drugs selectively inhibit viral DNA polymerases and have broad spectrum anti-herpes virus activity. Both lytic and latent herpes virus may be important in progression of PF. Antivirals target only lytic virus, but in doing so, indirectly reduce levels of latent virus by reducing ongoing infection of susceptible cells (81).

Despite our inability to cure latent herpes virus, or to prevent infection by vaccination, several animal studies have demonstrated clinical benefit from antivirals. Cidofovir administered to γ-herpes virus-infected IFNγR deficient (Th2-biased) mice, reduced the detection of herpes-related protein in lung and spleen, prevented progression of fibrosis and loss of lung function, and reduced production of TGFβ, inflammatory cytokines IFNγ, TNFα, and Th2 cytokines IL5 and IL13 (81). Treatment given early could prevent onset of pulmonary symptoms, but was beneficial in reducing mortality even after onset of symptomatic pulmonary fibrosis, although established fibrosis was not reversed. Interestingly, the associated rise in VEGF levels was controlled by early but not by late treatment. Cidofovir showed no benefit when PF was induced by bleomycin rather than by viral infection (81). Cidofovir was also effective in blocking CMV reactivation, attenuating the TNFα response and preventing PF in a caecal-puncture sepsis model (87).

Evidence of clinical benefit has also been demonstrated in humans. A patient with pulmonary fibrosis and a family history of rapid progression experienced clearance of pulmonary EBV expression, as measured by sputum PCR viral load and stabilization of lung function over 9 months, while on valaciclovir. A second patient treated with valaciclovir experienced clinical deterioration. However, this patient continued to have detectable sputum HHV7, despite EBV clearance (15).

In a small open-label study of 2 weeks' intravenous ganciclovir, 9 of 14 patients were classified as “responders” with improvement in a composite outcome of FVC, DTPA clearance, shuttle-walk test and reduction in prednisolone dose. Ganciclovir was well-tolerated without significant adverse events. Of those treated, 3 were alive without transplant, 2 had died and 3 had had lung transplant at 12 months follow up, compared with “non-responders” all of whom had died (3) or were post-transplant (2 subjects) (118).

Interferon gamma also has antiviral properties. In human PF and normal lung fibroblast cultures, IFNγ downregulates proliferation and differentiation to myofibroblasts in response to TGFβ and to act in a synergistic manner with pirfenidone (110). Although studies of subcutaneous IFN in established PF showed no benefit, a pilot study of inhaled IFN showed sustained reversal of DLCO (diffusing capacity of the lung for carbon monoxide) decline when administered to PF patients over a period of 80 weeks (119).

### Optimal Response to Antifibrotic Treatments May Depend on Herpes Virus Control

Applying learning from mouse models to treatment of patients, the anti-fibrotic effect of inhibition of TGFβ-ALK5 signaling in bleomycin-induced pulmonary fibrosis in mice is attenuated in the presence of concurrent γ-herpes virus infection, suggesting that herpes virus control may be necessary in order to optimize benefit from licensed antifibrotic drugs such as Pirfenidone. Pirfenidone may act by inhibiting TGFβ, probably indirectly, and, in the absence of pharmacological virus control, could potentially enhance herpes virus burden via the immunosuppressive effect of TGFβ (120).

### Diagnosis of Significant Herpes Virus Infection

Given the high community prevalence of herpes virus infections, the significance of current diagnostic tests, whether serological, antigen-based or histological, in a patient with PF, is largely unknown.

Better identification of biomarkers for relevant herpes virus infection may enable targeting of treatment to those most likely to benefit, and along with other biomarker-targeted treatment could enable truly personalized treatments. Animal studies clearly show that herpes virus-associated changes occur prior to onset of clinical features and in some cases that early treatment provides more greater benefit than later (81). For this reason, it will be all the more important to identify and diagnose people in the early stages of PF and to conduct trials at this time, rather than at symptomatic presentation, which is likely to represent a more treatment-resistant “end-stage” situation (11, 121, 122).

## Conclusion

Pulmonary fibrosis is an end-stage manifestation of many different and interacting processes. Although human and animal data suggests that herpes viruses are implicated in initiation, progression and exacerbation of pulmonary fibrosis, other studies suggest no such effect. Direct alveolar epithelial damage, induction of a profibrotic immunological environment, endoplasmic reticular stress, telomere attrition and cellular senescence are consequences of herpes virus infection, which, in concert with other environmental and genetic factors could increase the risk of pulmonary fibrosis. Techniques such as single cell RNA sequencing will provide important answers as to whether herpes viruses are implicated in pulmonary fibrosis. Cell specific data are required to understand the mechanisms by which herpes viruses could induce, maintain or exacerbate the fibrotic process. If herpes viruses are indeed relevant to pulmonary fibrosis, anti-herpes virus treatments could play a part in modifying or slowing pulmonary fibrosis progression, especially when used at an early stage.

## Data Availability Statement

The original contributions presented in the study are included in the article/supplementary material, further inquiries can be directed to the corresponding author/s.

## Author Contributions

AD conceived the paper and together with HL wrote the first draft. CS and JP reviewed the manuscript and contributed new sections. AD and JP compiled the tables. All authors contributed to the literature review and approve the final version.

## Conflict of Interest

The authors declare that the research was conducted in the absence of any commercial or financial relationships that could be construed as a potential conflict of interest.
